# Contribution of Polymers to Electronic Memory Devices and Applications

**DOI:** 10.3390/polym13213774

**Published:** 2021-10-31

**Authors:** Subin Lee, Somi Kim, Hocheon Yoo

**Affiliations:** Department of Electronic Engineering, Gachon University, Seongnam 1342, Korea; bini0211@gachon.ac.kr (S.L.); soomee123@gachon.ac.kr (S.K.)

**Keywords:** organic memory, floating gate, electret, ferroelectric, memristor, resistive memory, optoelectric, neuromorphic, flexible, biodegradable

## Abstract

Electronic memory devices, such as memristors, charge trap memory, and floating-gate memory, have been developed over the last decade. The use of polymers in electronic memory devices enables new opportunities, including easy-to-fabricate processes, mechanical flexibility, and neuromorphic applications. This review revisits recent efforts on polymer-based electronic memory developments. The versatile contributions of polymers for emerging memory devices are classified, providing a timely overview of such unconventional functionalities with a strong emphasis on the merits of polymer utilization. Furthermore, this review discusses the opportunities and challenges of polymer-based memory devices with respect to their device performance and stability for practical applications.

## 1. Introduction

Electronics using conventional silicon have been developed and are the core of various forms of modern electronic circuitry. On the other hand, an emerging category of electronic devices using polymers has attracted considerable attention, and extensive efforts have been made for the practical applications of electronic devices [1]. Owing to their solution-processability [2], easy-to-fabricate large-area deposition [3,4], and endurance to mechanical stress [5,6], polymers are considered promising candidate materials for functional devices, such as flexible and wearable thin-film transistors (TFTs) for next-generation soft display products [7] and disposable electronic sensors enabled by a low-cost fabrication process [8].

Among various electronic devices, memory devices, such as flash memory [9,10,11], dynamic random-access memory (DRAM) [11,12], and static random-access memory (SRAM) [13,14], have permeated most applications. Over the past 15 years, efforts have been directed towards the development of functional memory implementation using polymers as critical parts in various memory devices, including floating-gate memory [15,16], ferroelectric memory [17], filament-induced memristors [18,19], and charge-trapping memory [20,21]. Besides that, typical memory operations such as retention and memory switching, new functionalities have been reported in polymer-based memory devices. Optoelectrical switching behaviors, synapse-like operation, embedded memory on flexible/stretchable substrates, and biodegradable memory devices were recently developed, presenting the next-generation memory electronic applications.

This paper provides a timely overview of polymer-based memory devices. This review outlines the clear contribution of polymers to memory devices as a promising new field of investigation. Section 2.1 and Section 2.2 revisit the recent advances in floating-gate and polymer-electret memory devices, focusing on their fundamental operation behaviors and potential applications. Section 2.3 introduces two- and three-terminal ferroelectric memory devices, focusing on the role of polymers. Section 2.4 reviews the recent progress in polymer-based memristors, emphasizing the advantages of using a polymer as the active layer. Section 3.1 introduces the new memory functionality of the optoelectrical memory operation enabled by polymer use in memory devices. Section 3.2 reports a neuromorphic system demonstration using polymer-based memory devices as an emerging application. Section 3.3 introduces mechanically flexible memory integrations using polymers as another polymer-based memory direction. Section 3.4 introduces eco-friendly memory devices using biodegradable polymers.

## 2. Polymer-Based Memory Devices

### 2.1. Floating-Gate Memory

Floating-gate using polymers can be classified in various ways according to the material and operating mechanism. Depending on the material, the gates can be divided into organic memory using a metal-floating gate [16,22], nanoparticle polymer floating-gate [23,24], blended polymer [25,26], and nanocrystal floating-gate [27,28].

**Figure 1:**
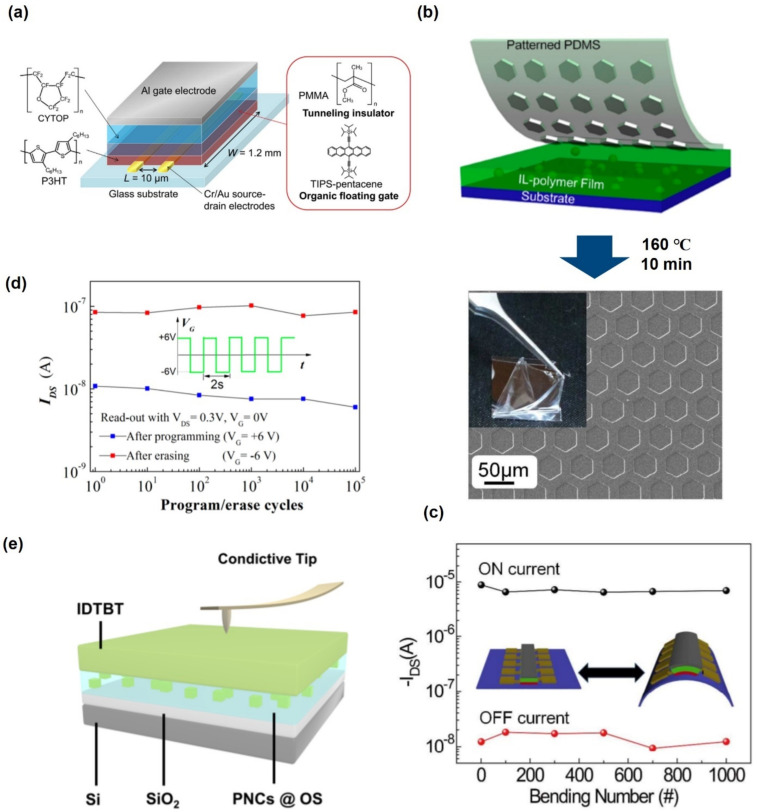
Classification of the floating-gate according to material: (**a**) metal electrode and using an organic semiconductor (adapted from [32] with permission from Elsevier.), (**b**) schematic and SEM image of the blended polymer (adapted from [33] with permission Figure 33. with permission from the American Chemical Society), (**c**) on, off current at using blended polymer (with permission from the American Chemical Society), (**d**) on/off current at the metal oxide nanoparticle (adapted from [34] with permission from the American Chemical Society), and (**e**) the schematic of floating-gate memory using nanocrystal (adapted from [35] with permission from the American Chemical Society).

Metal-floating gates in organic memory devices consist of a floating gate and organic semiconductor. Liu et al. reported a metal floating-gate using poly(4-vinylphenol) (PVP) [16]. A polymer layer of PVP was inserted between the electrode and the channel, where it worked as a tunneling barrier. The on/off switching current ratio was 1500 because the insulating characteristics of ultrathin PVP film cause hysteresis. Baeg et al. used gold as a single metal between the two organics of poly(4-vinyl-phenol (cPVP) and poly[9,9-dioctylfluorenyl-2,7-diyl]-co-(bithiophene)) (F8T2) (Figure 1a) [29]. The optimal layer (22 nm) of the F8T2 floating-gate, compared to the thick (25 nm) or thin (10 nm) layer, prevented metal penetration and increased mobility. Therefore, the on/off switching current ratio increases to more than 10^4^. Zhang et al. made a nano-floating gate using Ag-Pt bimetallic nanoparticles to improve the performance of single metal devices [30]. Bimetal showed better performance than each single device. Ag and Pt nanoparticles tend to trap holes and electrons, respectively. The floating gate in this device was made by depositing pentacene. The polymer shifted the threshold voltage (Vth) as the trap density increased and the device performance decreased. The on/off ratio showed more than 10^5^. On the other hand, maintaining a vacuum state in the manufacturing process brings a complex-to-fabricate process. Hence, as an alternative, Kim et al. reported a memory using reduced graphene oxide (rGO) as a floating gate and inkjet-printed electrodes of poly(3,4-ethylenethiophene)-poly(styrenesulfonate) (PEDOT:PSS) [31]. When a positive voltage is biased, the state becomes “programming” by electron tunneling rGO through the PMMA layer. When a negative voltage is biased, the state becomes “erase” by electron detrapping at the rGO layer, and the electron is transferred to the semiconductor layer again. Hence, they achieved an on/off switching current ratio of 10^4^ in the standby state using the inkjet method and developed a flexible device that can be scaled down. When a polymer is used as a dielectric layer, the operating voltage is quite high (Table 1). It is required to utilize and develop a thin and conformal insulating polymer coating technology for lower floating-gate voltage operation.

On the other hand, studies using quantum dot materials as floating gates have also been reported. Kim et al. fabricated the photo floating-gate memory using S-(3-mercaptopropyl) 2,3,4,5,6-pentafluorobenzothiote (FBC3SH) with a quantum dot (QDs) material CdSe, which was operated by an external stimulus [36]. The FBC3SH layer used as a tunneling insulator not only helps the surface modification to make the semiconductor layer grow well but also causes the device to turn on by accumulating holes by a large dipole moment. The memory device showed a stable and reliable current state at V_READ_ = 0 V (on state) during the device operation by synthesizing perfluorinated thiol ligand. Despite the large gaps between the grains of C10-DNTT deposited on the CdSe QD, OFETs with FBC3SH exhibited a linear current increasing trend even under V_GS_ = 0 V. The blended CdSe FBC3SH layers generated a large dipole moment when exposed to light, which altered the work function of the floating gate surface and induced the accumulation of hole carriers [37]. A quantum dot-based floating-gate allows the devices to exhibit large memory retention and high memory speed [38]. Li et al. fabricated ternary hybrid floating-gate memory using polystyrene (PS), [6,6]-phenyl-C61-butyric acid methyl ester (PCBM), which is used for hole trapping, and CsPbBr3 quantum dots (QDs), which trap photoinduced electrons [39]. This hybrid film exhibited ambipolar properties that store electrons and hole carriers using irradiating light and a biasing voltage, respectively. As a result, it showed a fast-erasing speed of 0.05 s and a low erasing voltage of −35 V. This dual-functional memory device may provide a new future for memory devices.

Nanoparticle (NP) floating-gates can be adjusted to optimize memory performance by their size, density, and assembly. Sun et al. fabricated an ambipolar floating gate by mixing Au NP with PMMA used as the tunneling dielectric [40]. Owing to the ambipolar characteristics, a memory window of 20 V and an on/off switching current ratio of 10^4^ were achieved. Zhang et al. reported a PS floating gate blended with Ag and Pt NPs [41]. The holes in a bimetal-based floating-gate device, in which two materials are used in one device, were operated using the Fowler–Nordheim tunneling method. At PS floating gate layer, Ag and Pt NPs, traps holes and electrons, respectively. Using the bimetal device, the memory window was 18.7 V, which was approximately 10 V higher than the single metal. The on/off ratio showed a value of more than 10^5^.

The tunneling dielectrics of memory fabricated by blending two or more polymers have also been reported. Wu et al. used blended 6, 13-bis-(triisopropylsilylethynyl) pentacene (TIPS-Pentacene) and polymer poly(styrene) (PS) [41,42]. The blended material processed by solution acted as a floating-gate and tunneling layer. A large memory window of 25 V or more and a stable cycle of more than 500 cycles were obtained by optimizing the proportion of TIPS-Pentacene and PS.

When a gate voltage is applied to the conventional floating-gate memory, carriers are trapped and detrapped in the floating gate, causing device operation. In contrast to the mentioned conventional operation, this section revisits an emerging photo-assisted memory operation that uses the photoelectric effect to generate an electric field by irradiating light without a biasing voltage.

Chen et al. used poly(2,7-(9,9-dioctylfluorene)) (PFO) and polystyrene (PS) blended according to an appropriate ratio to use PFO5k-b-PS22k as a floating gate [43]. The formation enhanced electron trapping, and the stabilized electrons were trapped at blended PFO and PS interfaces. The device showed an on/off switching ratio of more than 10^4^, and it was stable for more than 10,000 s. Yang et al. used a photoactive material, CH_3_NH_3_PbBr_3_/poly(2-vinylpyridine), as a floating gate [44]. The gate was most active at 530 nm and was divided into photo-writing, reading, photo-erasing, and reading cycles by light irradiating. It showed an on/off ratio of more than 10^4^, and photo-erasing was possible only with light irradiation only for one second. In addition, the substrate was a flexible device using the poly(ethylene 2,6-naphthalene dicarboxylate) (PEN) film and showed the same photoactivity after 1000 bending cycles. A memory that writes at 675 nm was reported. Lan et al. showed used a blended polymer indacenodithiophene-benzothiadiazol (IDTBT) and poly[1,6-bis(dicarboximide)-2,6-diayl] (N2200) to fabricate an ambipolar memory that served as a floating-gate [45]. Quantum well-like organic heterojunctions act as charge trap centers in the n-type material. The heterojunction’s stored optical information expanded it to an 8X8 active-matrix array. The aforementioned results suggested a path for a photosensor with a new mechanism of image sensing.

Optical devices can repeat the erasing-reading-programming process when light is irradiated. Photoerasable devices are unlike the device that reads first. Li et al. used a floating-gate by blending [6,6]-phenyl-C61-butyric acid methyl ester (PCBM) and PMMA materials. They stored 2 bits by trapping electron carriers even under various bias conditions. As erasing was first, it showed memory that can erase mass data [46]. Jeong et al. reported floating-gate memory using QD type CdSe and pentacene [47]. Surface-modified “photoinduced recovery” is possible in a device that is erased first, and small molecular ligands induce hole diffusion from the QD to the conductive channel, resulting in a fast erasing time. The surface-modified QDs with fluorinated molecules function as normally-ON type transistor memories with a non-destructive operation, showing a stable current state and an on/off switching ratio of more than 10^5^.

**Figure 2:**
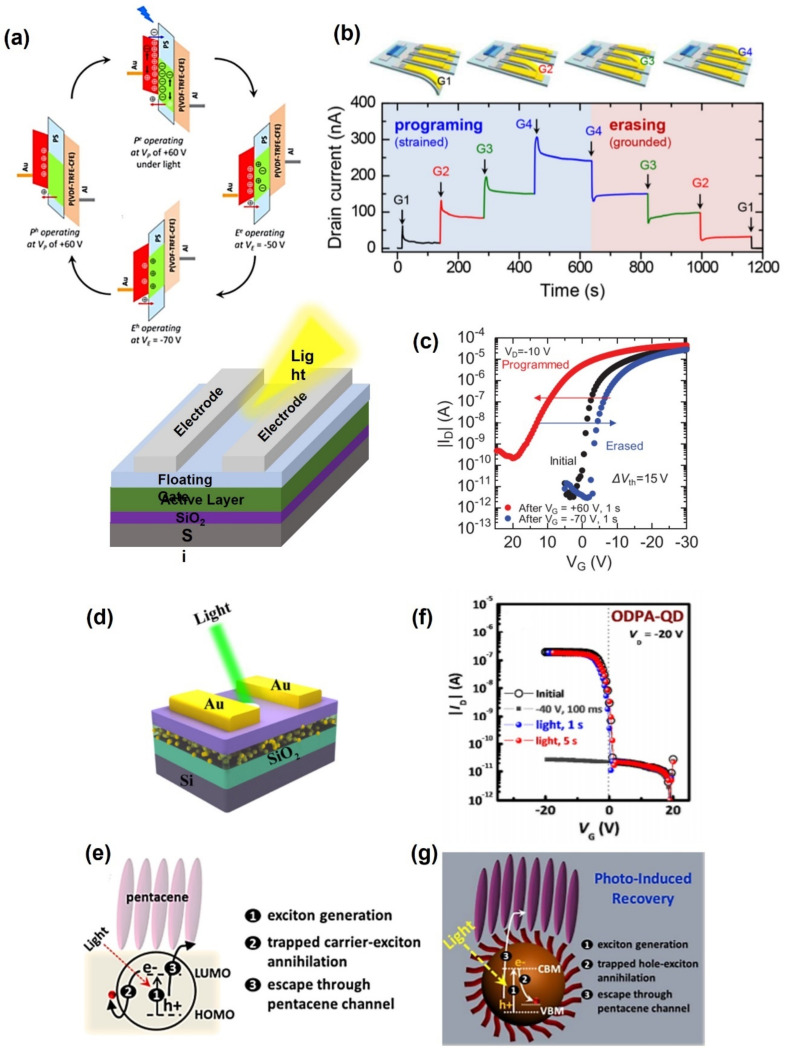
Working mechanisms using polymer floating-gate memory with (**a**) three-level (adapted from [50] with permission from the American Chemical Society) and (**b**) four-level information storage (adapted from [51] with permission from the American Chemical Society); accommodate memory level programming and erasing (**c**) using a photo (adapted from [41] with permission from AIP Publishing) and (**d**) a schematic diagram of photo writes (adapted from [46] with permission from the American Chemical Society); (**e**) principle of photo writers (adapted from [47] with permission from the American Chemical Society); (**f**) a graph of photo erasable memory (adapted from [52] with permission from the American Chemical Society) and (**g**) its principle according to energy bandgap (adapted from [52] with permission from the American Chemical Society).

A mechanism using the photoelectric effect does not store two levels on the 0, 1 state, but a multilevel was reported. Xu et al. fabricated a polymer floating-gate using poly(9,9-dioctylfluorene-co-benzothiadiazole) (F8BT) and polystyrene. With suitable optical and electrical programming/erasing conditions, it became a three-level information storage state. The electronic storage of the floating gate, approximate neutrality, and hole storage enabled three levels of information storage (Figure 2a). Jin et al. suggested a non-volatile memory device with four levels of information storage [48]. The Cs_2_Pb(SCN)_2_Br_2_/polymer (CPB) was used as a floating gate, and light with an absorption wavelength of 470 nm according to CPB QD was used. The stored electrons were depleted at the floating gate, and the concentration of holes in the conductive channel increased, and the transfer curve shifted to the initial state. The floating-gate organic field-effect transistor memory (FGOFETM) has a very stable data retention function, as the applied wavelength and the current level are maintained at the same value for 20 cycles depending on the applied voltage [48]. Furthermore, Zhou et al. prepared an ambipolar memory using poly(diketopyrrolopyrrole-thiophenebenzothiadiazolethiophene) (PDPP-TBT) as a floating gate. Charge trapping occurred in the metal nanoparticle, trapping both electrons and holes [49]. The device had a wide memory window, and five levels were separated, suggesting a multibit flash memory.

**Table 1:** Polymer-based floating-gate memory performance comparison.

	Polymer	Thickness (nm)	On/Off Switching Ratio	Memory Window (V)	Retention Time (s)	Mobility (cm^2^ V^−1^ s^−1^)	[Ref]
Floating gate	cPVP/F8T2	22	>10^4^	30	a few hours	0.02	[29]
	FBC3SH	3.67	<10^2^	18.7	10^5^	0.05	[37]
	PCBM/CsPbBr_3_/PS	62	10^2^	12.5	10^4^	N/A	[39]
	TIPS Pentacene	45	10^2^	>25	5000	0.065	[42]
	PFO/PS	3.48	10^4^	21	10^4^	0.0074	[43]
	CH_3_NH_3_PbBr_3_/poly(2-vinylpyridine)	60	<10^4^	14	>10^4^	N/A	[44]
	PCBM/PMMA	9.90	>10^2^	22.1	1.2 × 10^4^	N/A	[46]
	PVN/C_60_	0.3	10^5^–10^6^	10.31	10^4^	0.26	[47]
	F8BT/Polystyrene	50	10^6^	5, 17 (at each level state)	2 × 10^4^	1	[50]
Tunneling layer	PVP	16	1500	N/A	200	0.02	[16]
	Pentacene	10–20	>10^5^	18.7	10^4^	0.53	[30]
	PMMA	8–10	10^4^	20	N/A	N/A	[36]
	PMMA	100	10^2^	20	10^3^	0.15	[41]
Electrode	PEDOT:PSS	10	>10^4^	10	10^7^	0.08	[31]
Semiconductor	DPP-DTT	80	10^3^	20	10^8^	0.3	[42]

### 2.2. Polymer Electret Memory

**Figure 3:**
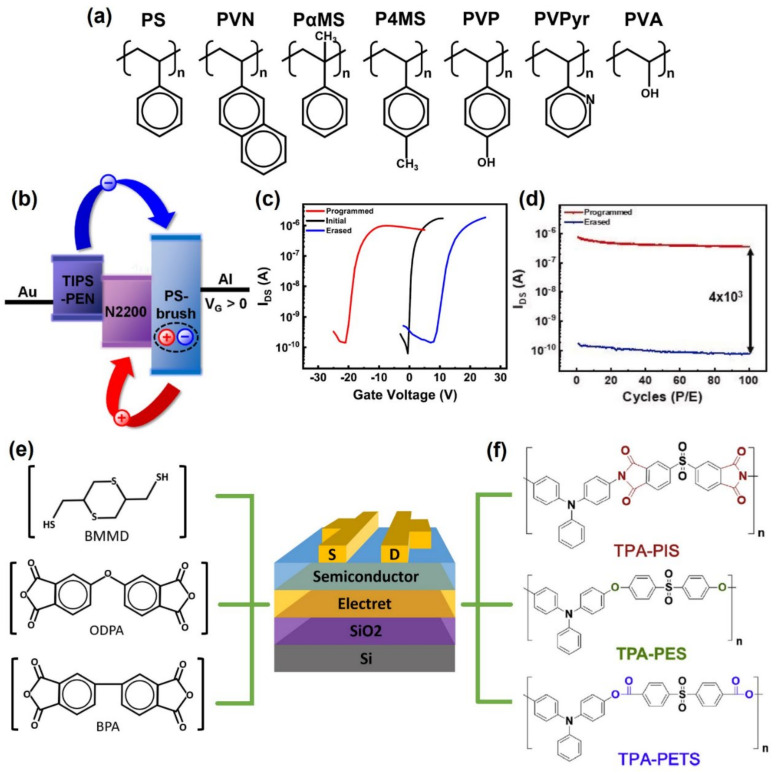
(**a**) Styline-based polymer electret materials; PS, PVN, PaMS, P4MS, PVP, PVPy, and PVA (adapted from [55] with permission from John Wiley and Son). (**b**) Mechanism, (**c**) current–voltage curve, (**d**) retention properties of polymer electret memory using styrene-based (polystyrene-brush) PS-brush electret(adapted from [56] with permission from Elsevier). Schematic configuration of the OFET memory device and chemical structures of the polymer electret layers: (**e**) PITE(BMI-BMMD), PI(APS-BPA), PI(APS-ODPA)) (adapted from [21] with permission from John Wiley and Son) (**f**) TPA-PIS, TPA-PES, and TPA-PET (adapted from [57] with permission from MDPI).

Similar to the operation of a floating gate, a polymer electret memory was also considered as another charge trapping memory. Polymer electret, which is a dielectric material with a quasi-permanent electrical charge, has been used widely in sensors, memories, and gas filters [53,54]. The memory device operates the storage of charges in the polymer electret layer. The following mechanisms can explain the storage of charges in the polymer electrets: orientation of permanent dipoles (in polar materials) and charge-trapping by structural defects or boundaries [55]. Kim et al. reported the properties of various polymer electrets based on styrene [55]. Polymer electret OFET memory implementations using styrene-based polymers, such as polystyrene (PS), poly(α-methylstyrene) (PaMS), poly(4-methyl styrene) (P4MS), poly(2-vinyl naphthalene) (PVN), poly(4-vinyl phenol) (PVP), poly(2-vinyl pyridine) (PVPyr), and polyvinyl alcohol (PVA), were performed (Figure 3a). Organic field-effect transistor devices using hydrophobic and non-polar polymers (PS, PaMS, and PVN) had better non-volatile memory properties than hydrophilic and polar polymers (PVA, PVP, and PVPyr). This phenomenon can be explained by that polar and hydrophilic polymer electrets were difficult to store electric charges because the trapped electrons were released rapidly by dipoles, moisture in the atmosphere, and ions frequently present in the hydrophilic polymer.

Recently, ambipolar characteristic memories that can store both carriers (i.e., electrons and holes) have been reported. He et al. reported the storage properties of poly(α-methylstyrene) (PαMS)-based ambipolar memory as the transport properties of minority carriers (electrons) in organic semiconductors (Pentacene) [58]. They fabricated a memory device using a PαMS electret layer. Electrons and holes were captured in the trap state of PαMS according to the gate bias to induce the memory characteristics. Ambipolar storage memory has several advantages, such as a large memory window, multilevel memory, and low-voltage operation [59]. Therefore, a 24 V memory window and 10^5^ of on/off switching current ratio were obtained. The retention characteristics of the memory were highlighted by the on/off ratio being maintained even after three hours. Kim et al. implemented an ambipolar device by blending the semiconductor layer with n-type N2200 and p-type TIPS-Pentacene (Figure 3b) [56]. The electret layer was manufactured using a styrene-based (polystyrene-brush) PS-brush electret. By applying blends of organic semiconductors with an optimized proportion ratio, the resulting memory devices showed a 30 V memory window with a 30 V operating voltage, stability endurance performance over 100 times, and 10^8^ s retention time while maintaining a 10^4^ on/off switching current ratio (Figure 3c,d).

Although polymer electret memories using styrene-based electret materials have been reported [60,61], electret memory devices fabricated from the organic polymer have also been reported. Yu et al. reported various electret n-type polymer electret FETs using a polyimide (PI) [21]. Polyimidothioether [4,40(diaminodiphenylsulfide) bismaleimide- 2,5-bis(mercaptomethyl)-1,4-dithiane] (PITE(BMI-BMMD)), poly- [bis-(4-aminophenyl)-sulfide-biphthalimide] (PI(APS-BPA)), and poly[bis-(4aminophenyl)-sulfide-oxydiphthalimide] (PI(APS-ODPA)) polymer electret layers were synthesized using 2,5-Bis(mercaptomethyl)-1,4-dithiane (BMMD), 4,4′-oxydiphthalic anhydride (ODPA), and 4,4′-biphthalic anhydride (BPA) (Figure 3e). Although all three electret layers were synthesized using PI, the synthesized PI electret layers had different charge transfer functions. Therefore, PI(APS-ODPA) and PI(APS-BPA) memory operate as non-volatile flash memory capable of four states of write-read-erase-read (WRER), but PITE(BMI-BMMD) memory operated nonvolatile write-one-read-many (WORM) memory characteristics. Therefore, transistor memory devices can be operated by controlling the charge transfer function of the electret. Cheng et al. implemented three polymer electret layers of TPA-PIS, TPA-PES, and TPA-PETS by combining polyimide (PI), polyether (PE), and polyester (PET) with triphenylamine (TPA) (Figure 3f) [57]. TPA-PIS and TPA-PETS electret memory devices exhibited ambipolar charge trapping behavior, resulting in a memory window of 32.4 ± 1.2 and 21.7 ± 1.0 V and an on/off switching current ratio of 10^5^~10^6^ and 10^3^~10^4^, respectively. The TPA-PES device exhibited unipolar hole trapping properties because of the high LUMO level and the large barrier offset with pentacene. While on/off switching ratio and retention time performances are excellent in the reported polymer electret memory (Table 2), the programming/erasing voltage values are quite high due to the thick polymers. Efforts to make thinner polymer electrets should be performed for lower voltage operations.

### 2.3. Ferroelectric Memory

#### 2.3.1. Two-Terminal Ferroelectric Memory

**Figure 4:**
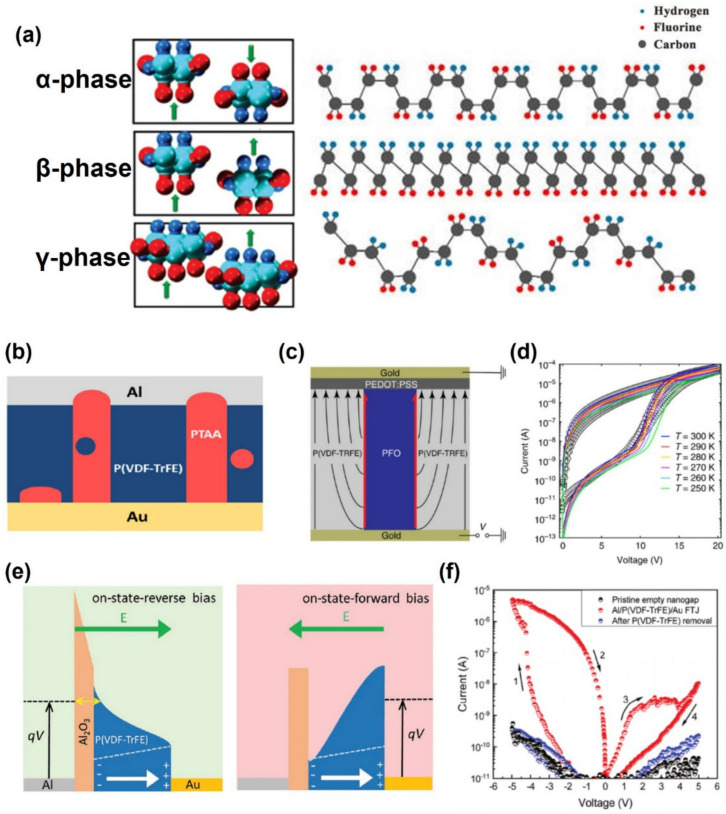
(**a**) Illustration of the crystalline phase of PVDF (adapted from [69] with permission from MDPI and [68] with permission from American Chemical Society). (**b**–**d**) Diode type ferroelectric memory and characteristics (adapted from [74] with permission from Nature and [75] with permission from the American Institute of Physics). (**e**,**f**) Mechanism and transfer curve of FTJ type ferroelectric memory (adapted from [76] with permission from John Wiley and Son).

When electricity is applied to a ferroelectric material, it exhibits magnetism that maintains electric polarization by aligning dipoles. The aligned dipole polarization can be reversed by an electric field that is larger than the coercive field and generate stored data ‘0’ and ‘1’ [62,63]. As a result, ferroelectricity provides non-volatile memory operation, in which recorded data is not erased, even when the power is turned off [64,65]. Ferroelectric memory will be needed in the era of the Internet of Things (IoT), owing to its high speed and low power consumption [66,67]. Recently, the most commonly used and studied ferroelectric polymer is polyvinylidene fluoride (PVDF), whose molecular repeating unit is based on a CH_2_-CF_2_ vinylidene (VDF) monomer with two fluorine and two hydrogen atoms bonded to a carbon backbone. PVDF has four complicated crystalline structures: α, β, and γ, as shown in Figure 4a [68,69]. Because the α phase remains very thermally stable, the PVDF solutions usually crystallize as non-ferroelectric α phases because the α phase remains very thermally stable and has lower potential energy of −6.03 kcal/monomer unit than the −5.73 kcal/monomer unit of the β phase. In contrast, the β and γ phases are polarized and exhibit ferroelectric properties because they are composed of all trans molecules packaged in a parallel manner. Among these, the β phase exhibits the largest polarization, 13 μC/cm^2^ [70].

Poly(vinylidenefluoride-co-trifluoroethylene) (P(VDF-TrFE)) is the most common and widely used ferroelectric polymer using PVDF [71,72]. P(VDF-TrFE) realizes robust polarization effect using simple methods, such as spin coating and roll-to-roll-based inkjet printing technology without a thermal budget [73]. This Section introduces two-terminal resistance switching ferroelectric memory as three mechanisms: (1) filamentary resistive switching type, (2) diode type, and (3) ferroelectric tunnel junctions (FTJs) type.

First, Kim et al. implemented an organic ferroelectric resistive random-access switching memory using ZnO nanoparticles (NP) and P(VDF-TrFE), which has the ferroelectric polarization effect [77]. As the applied voltage was increased, the ZnO nanocomposite was aligned in the direction of the dipole field of the ferroelectric to form a filament. Among the various mixing ratios, the devices were fabricated using the optimal ratio of 20 wt. % ZnO nanoparticles exhibited optimal and stable resistive switching behavior with an on/off switching current ratio of up to 2 × 10^7^ and a holding time of 10^4^ s at a low operating voltage (−4 V~4 V). There are also reports of the implementation of resistive switching memories by controlling the space charge effect through defects in the ferroelectric film.

Second, diode-type devices have been developed as an alternative because it is difficult to form a tunneling current or a filament due to a thick ferroelectric layer. Therefore, the ferroelectric diode has a mechanism in which carriers are transported by ferroelectric polarization in the patterned semiconductor, as shown in Figure 4c [68,75]. Lenz et al. produced a ferroelectric diode using the polymer poly(9,9-dioctylfluorene) (PFO) as the semiconductor and P(VDF-TrFE) as the ferroelectric polymer by the soft lithography method [78]. When the positive bias increased, it changed to efficient charge injection such as ohmic metal–semiconductor contact by ferroelectric P(VDF-TrFE) polarization compared to Schottky contact at non-polarization. In addition, a large amount of charge transport occurs at the semiconductor–ferroelectric interface because of the strong charge accumulation. Therefore, the diode showed an on state because of the resulting set and reset. At this time, the diode achieved an on-current density greater than 10^3^ A/m^2^ that was maintained for more than 10^4^ s with an on/off switching current ratio of 10^3^.

Finally, ferroelectric tunnel junctions (FTJs) are considered outstanding candidates of memory devices for low-power nonvolatile memory and memristic devices [79]. Ferroelectric polarization switching alters the ferroelectric–electrode interface barrier height, thereby changing the tunneling electron resistance (TER) of the FTJs, modulating the current at the metal/thin-film ferroelectric/metal sandwich structure. A higher TER ratio results in greater hysteresis through polarization reorientation of the ferroelectric tunnel barrier, maintaining the two stable nonvolatile states. Majumdar et al. altered the annealing conditions of P(VDF-TrFE) ultrathin films in the structure of Au/P(VDF-TrFE)/Nb-doped SrTiO_3_(NSTO) FTJs and reported the switching time of the FTJs [73]. They demonstrated the higher the crystallinity by changing the annealing temperature of ferroelectric, the better the switching characteristics. In addition, large TER was generated by changing the barrier width caused by electron tunneling by biasing. Therefore, P(VDF-TrFE) showed a switching speed of 1 ps to 1 ns, which is comparable to ceramic ferroelectrics, such as BTO or PZT (about 100 ps–10 ns). Kumar et al. reported FTJs based on a ferroelectric polymer P(VDF-TrFE) using adhesion lithography to fabricate nanogaps electrodes and showed stable and reproducible large TERs [76]. The ferroelectric polarization of P(VDF-TrFE) caused the FTJ tunneling current modulation. When forward biased, the Al-oxide barrier acted as an additional barrier to charge transport because the ferroelectric polarization and the applied external field were in reverse equilibrium (Figure 4e). Therefore, the barrier modulation of the Al/P(VDF-TrFE) electrode interface showed a huge effect of 106% in the planar FTJ.

#### 2.3.2. Three-Terminal Ferroelectric Memory

Unlike the two-terminal ferroelectric memory, the three-terminal ferroelectric memory exhibits field-effect transistor (FET) formation (Table 3). Most reported ferroelectric field-effect transistors (FeFET) used ferroelectric materials as gate insulators. A FeFET and general FET has the same operating mechanism, but FeFET is affected by ferroelectric polarization. Assuming that the semiconductor and gate insulator are n-type, the FeFET memory operates as follows. (1) At positive voltage biasing at the gate, when the polarization direction is toward the semiconductor channel, electrons are accumulated at the semiconductor–ferroelectric interface to form a channel, and the current flows at electrodes. (2) When 0 V is biased at the gate (Vgs = 0 V), FeFETs are still polarized, so the channel does not disappear, and the current flows easily, unlike typical FETs. Therefore, (3) a negative voltage must be applied to alter the polarization direction and block the channel. By changing the polarization direction, two states of 0 and 1 are realized because of hysteresis. Ferroelectric polarization allows the transistor to maintain a state of on or off without additional biasing. Therefore, FeFET can be used, such as data loss-free and non-volatile memory [80].

The ferroelectric film of FeFET is a crucial part of interfacing with semiconductors. If the ferroelectric layer and the semiconductor or electrode interface are bonded unsuitably, the polarization characteristics of the ferroelectric do not occur, the leakage current increases due to carrier trapping, and a large operating voltage is required [81]. Therefore, various studies have improved the performance of the FeFET, such as using a buffer layer and improving the surface roughness of the ferroelectric film and the interface with the semiconductor. This paper reports FeFETs using various semiconductors layers: organic materials, 2D materials, and metal oxides.

**Figure 5:**
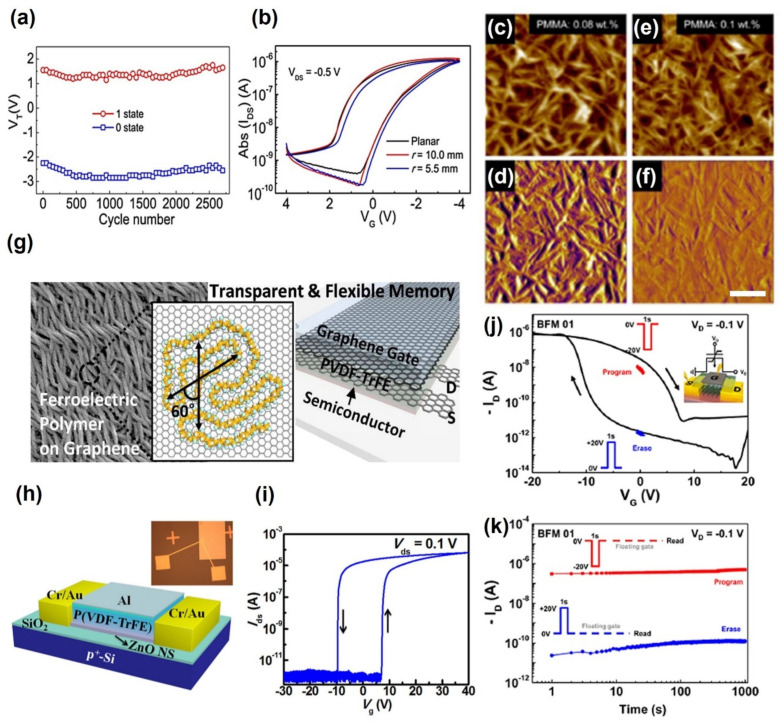
(**a**,**b**) Stable data storage retention over 8 × 10^4^ s with a memory 10^2^ of on-off in P(VDF-TrFE-CTFE) ferroelectric FETs (adapted from [84] with permission from Nature). (**c**–**f**) RMS roughness of the PMMA/P(VDF-TrFE) surface with increasing PMMA concentration from 0.08 wt. % to 0.1 wt. % (adapted from [86] with permission from the American Chemical Society). (**g**) Ferroelectric FETs using 2D materials; graphene (adapted from [87] with permission from the American Chemical Society) and (**j**,**k**) black phosphorus (BP) (adapted from [88] with permission from the American Chemical Society). (**h**,**i**) Structural and I–V characteristic curve of ferroelectric FETs using metal oxide (ZnO) (adapted from [89] with permission from AIP Publishing).

Nguyen et al. fabricated non-volatile FeFET memory using pentacene as a semiconductor layer and P(VDF-TrFE) as a gate insulator [82]. They improved the surface roughness by changing the crystallinity of the P(VDF-TrFE) film by annealing at 140 °C. In addition, a 10^4^ of on/off switching current ratio was maintained for 5000 s. On the other hand, the performance of PEN-based organic FeFETs is affected by a range of conditions causing a leakage current [83]. Therefore, to prevent the leakage current, Xu et al. proposed a novel structure to overcome the leakage current using a ferroelectric polymer between two-buffer ultrathin AlO_X_ [84]. By inserting the AlO_X_ layers, large mobility of 1.7~3.3 cm^2^ V^−1^s^−1^, highly reliable memory switching endurance of more than 2700 cycles, and high stable data storage retention of more than 8 × 10^4^ s with a memory on-off switching ratio of more than 10^2^ were presented (Figure 5a,b). On the other hand, Xu et al. deposited a long-chain alkane molecule tetratetracontane (TTC) as a protective layer on poly(vinylidenefluoride–trifluoroethylene–chlorotrifluoroethylene) terpolymers (P(VDF-TrFE-CTFE)) film surface to protect the interfacial trap and improve the pentacene film crystalline quality [85] Therefore, FeOFETs non-volatile memory reported an operation voltage of ±15 V, mobility up to 0.5 cm^2^ V^−1^s^−1^, and stable program/erase over 1000 cycles. They achieved storage retention and durability of 6000 s. In addition to pentacene, other organic materials have been used. Song et al. presented FeFETs using C8-BTBT as a semiconductor layer and PMMA as a buffer layer [86]. The root mean square (RMS) roughness of the PMMA/P(VDF-TrFE) surface was reduced from 3.6 to 2.7 nm by increasing the PMMA concentration from 0.08 wt. % to 0.1 wt. % (Figure 5c–f). Thus, carrier mobility up to 5.6 cm^2^ V^−1^s^−1^ and the on/off ratio of 10^6^ can be achieved. The device exhibits switching times of ~3.0 ms from off to on.

FeFETs made from semiconductor layers using 2D materials have also been reported. A 2D semiconductor layer has a large memory window because of its high conductivity and ambipolar properties. Amiri et al. fabricated a top gate structure FeFETs by depositing graphene [90]. They used the ambipolar property of graphene to fabricate FeFETs and revealed the hysteresis transfer properties with two distinct bistable conductivity levels at zero gate bias. They reported an on/off ratio of 2.4, contact resistance of 50 Ω, and mobility of 400 cm^2^ V^−1^s^−1^. Lee et al. fabricated black phosphorus (BP)-based nonvolatile memory FETs using P(VDF-TrFE) as the top gate insulator layer [88]. Among the 2D vdW materials, BP showed significant potential in electronic and optoelectronic applications because of its allotropic properties, high mobility, and direct and narrow bandgap [91,92]. Despite its different unipolar properties from graphene, the BP FeFETs exhibit a memory window of 15 V and a linear mobility value of 1159 cm^2^V^−1^s^−1^, with an on/off current ratio of 10^3^ at room temperature (Figure 5j). The device was held for 1000 s without degrading the program/erase ratio (Figure 5k).

In addition, there are cases where high-performance FeFETs are manufactured using zinc oxide (ZnO), a metal oxide. Tian et al. fabricated FeFETs using the synthesized ultra-thin zinc oxide nanosheets (ZnO NS) (Figure 5h) [89]. The P(VDF-TrFE) film protects the surface traps and defects of ZnO NS and blocks the absorption and desorption of air, which affects the conductivity of ZnO. Owing to the excellent residual polarization properties and high single crystal quality of the ZnO nanosheets, an on/off current ratio of more than 10^7^ and a large memory window of 16.9 V were obtained at a 0 V gate voltage and 0.1 V drain voltage, as shown in Figure 5i. In addition, the measured Program/Erase retention time was more than 3000 s.

FETs using a blended layer of ferroelectric and polymer to improve the performance were reported. Kim et al. fabricated a FeFET using a P(VDF-TrFE)/PMMA blended buffer layer to improve the electrical performance [93]. FeFETs with blended buffer bilayers (BL-FeFETs) showed a more than 25 times higher on-current (3.40 A) than single-layer FeFETs (SL-FeFETs) (130 nA). BL-FeFETs showed improved memory retention (10^3^ s) and a higher on/off switching current ratio than conventional SL-FeFETs (Table 3).

**Table 3:** Ferroelectric memory characteristics comparison.

	Ferroelectric Polymer	Thickness (nm)	On/Off Switching Ratio	Memory Window (V)	Retention Time (s)	Mobility (cm^2^ V^−1^ s^−1^)	[Ref]
2terminal ferroelectric layer (Switching layer)	P(VDF-TrFE)_ ZnO NPs blends	300	2 × 10^7^	N/A	10^4^	N/A	[77]
	P(VDF-TrFE)	300	10^3^	N/A	10^4^	N/A	[78]
	P(VDF-TrFE)	250 ± 54	10^2^	N/A	8.7 × 10^4^	N/A	[74]
	P(VDF-TrFE)	265 ± 10	10^5^	N/A	N/A	N/A	[75]
	P(VDF-TrFE)	6	10^2^–10^4^	N/A	10^9^	N/A	[73]
	P(VDF-TrFE)	300	10^5^	N/A	N/A	N/A	[76]
3terminal ferroelectric layer (Gate insulator)	P(VDF-TrFE)	900	10^4^	N/A	5 × 10^3^	0.072–0.12	[82]
	P(VDF-TrFE-CTFE)	60	10^2^	13.3	8 × 10^4^	1.7–3.3	[84]
	P(VDF-TrFE-CTFE)	650	10^3^–10^4^	12.4	6 × 10^3^	0.5	[85]
	P(VDF-TrFE)	300	10^6^	12 ± 2	10^6^	5.6	[86]
	P(VDF-TrFE)	800–1500	2.4	N/A	N/A	400	[90]
	P(VDF-TrFE)	220	10^3^	15	4 × 10^2^	1159	[88]
	P(VDF-TrFE)	300	10^7^	16.9	3 × 10^3^	588	[89]
	P(VDF-TrFE)_ PMMA blends	N/A	10^4^	N/A	10^3^	N/A	[93]

### 2.4. Memristor

A memristor is a device that shows a resistance switching effect in relation to the coupling between electric charge and magnetic flux. The structure is divided mainly into vertical and horizontal structures. The vertical structure is where the insulator material is sandwiched between two electrodes. The two electrodes are in the horizontal direction, and the carrier movement through the semiconductor material below is the horizontal structure. In both devices, the operation of negative ions is the main operation, and it shows the resistance switching effect exhibiting hysteresis by forming filaments in the polymer layer [94].

**Figure 6:**
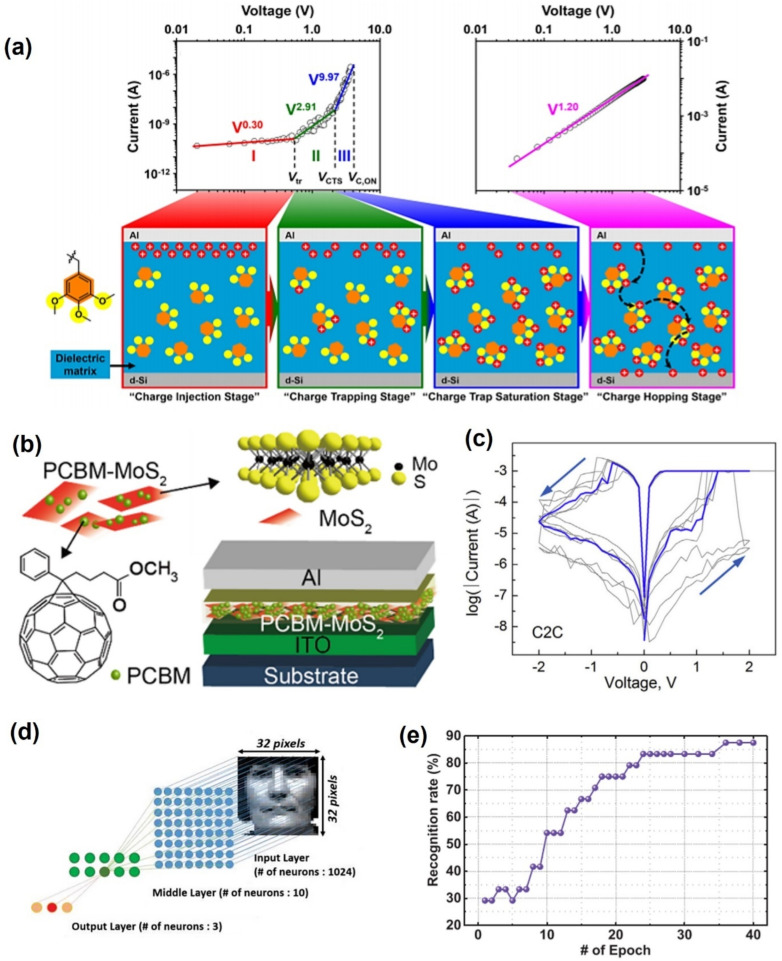
Classification of polymers according to the properties of the substances: (**a**) operation mechanism using oxygen-based polymers (adapted from [95] with permission from the American Chemical Society), (**b**) 2D material polymers (adapted from [97] with permission from the American Chemical Society), (**c**) conductive metal bridges (filaments) (adapted from [98] with permission from Elsevier), (**d**) realization memristor device using PVP (adapted from [99] with permission from the American Chemical Society), and (**e**) recognition rate by measuring the PVP memristor (adapted from [99] with permission from the American Chemical Society).

Ree et al. synthesized novel oxygen-based polymers through reacting poly(ethylene-alt-maleate) with oxybenzyl alcohol; poly(ethylene-alt-di(3,4,5-trimethoxybenzylmaleate) (PEM-BzOMe3) as the switching layer with the Al electrode [95]. Schottky emission and trap space charge limited conductions in the off state and hopping conduction in the on state at PEM-BzOMe3 12.2 nm in Figure 6a. This memristor exhibited p-type unipolar resistive memory behavior with low set voltages at 9.97 V, on/off switching current ratio of 10^8^, and high reliability for 40,000 s.

Several memristor devices using organic 2D material with an ordered structure, high surface area, high stability, and tunable function were also reported [96]. Lv et al. fabricated a memristor using a conventional diode with van der Waals heterostructures (vdWHs) using [6,6]-phenyl-C61-butyric acid methyl ester (PCBM)−molybdenum disulfide (MoS_2_) nanocomposites [97].

The vdWHs dominate the electric characteristics. The electronic characteristics of the diode devices were tuned by changing the surface deposition ratio of PCBM nanoaggregates on MoS_2_ nanosheets. PCBM can be polarized under the applied electrical field, resulting in the build-in localized internal electrical fields, even when the voltage is turned off, leading to the nonvolatile properties of the device. The device exhibited rewritable resistive switching with low switching voltages (~2 V), high current on/off ratios (~3 × 10^2^), and superior electrical bistability (>10^4^ s).

Minnekhanov et al. reported a sandwich memristor with Cu/Parylene dimer (2,2-para-cyclophane)/Indium tin oxide (ITO) structure [98]. When Cu is biased with a positive voltage, the reduced Cu metal ions are injected into the polymer layer to form a conductive filament, connecting the top and bottom electrodes. When a negative voltage is applied, the thinnest filament ruptures due to Joule heating, demonstrating the mechanism that results from the HRS state (Figure 6c). In addition, the conductance quantization effect was observed to confirm the switching, which will be useful in the development of multilevel data memory cells.

Chen et al. fabricated a flexible memristor by bonding parylene to nanopore graphene [100]. As mentioned earlier, a parylene-based device reduces the reset current and programming power consumption. Therefore, the intrinsic nanopores on graphene form conductive filaments at low currents. As a result, compared to the device without a graphene layer, the reset current was lowered 47 times, and programming power consumption was reduced 14 times.

There are memristors using the water-soluble polymer polyvinyllpyrrollidone (PVP). Jang et al. fabricated a memristor using poly(1,3,5-trivinyl-1,3,5-trimethyl cyclotrisiloxane) (pV3D3) as the interlayer using two Cu and Al electrodes [99]. The uniform, pinhole-free film pV3D3 allowed the stable formation of Cu metal ions between the top and bottom electrodes. In addition, as pV3D3 was controlled by the voltage, synaptic switching behavior was realized. Flexible devices can be fabricated because of the properties of the material. To implement practical applications based on polymer memristors, robust endurance should be developed, which is still lacking in the reported studies (Table 4).

**Table 4:** Memristor characteristics comparison.

	Polymer	Thickness (nm)	On/Off Switching Ratio	Memory Window (V)	Retention Time (s)	Mobility (cm^2^ V^−1^ s^−1^)	[Ref]
Switching Layer	PEM-BzOMe_3_	35.6	10^8^	4	4 × 10^4^	N/A	[95]
	PCBM-MoS_2_	50	3 × 10^2^	4.5	>10^4^	N/A	[97]
	Parylene dimer(2,2-para-cycloophane)	100	10^3^	4	>10^4^	N/A	[98]
	Parylene	40	10^5^	2.5	10^4^	N/A	[100]
	pV3D3	13.5	10^5^	<1	>10^3^	N/A	[99]

## 3. New Applications

### 3.1. Optoelectrical Memory

The light device performance can be improved without damaging the device, and it can be an independent variable to control the device. When light is irradiated on the photoreactive material, the electrical signal becomes an additional electric field by the generated photons. Thus, several types of memory, such as field-effect transistor memory, resistive random-access memory, multilevel memory, and phase change memory, have used photo-generated carriers to improve the performance. Therefore, many optically variable memories with unique characteristics using a range of photoreactive materials have been reported (Table 5).

**Figure 7:**
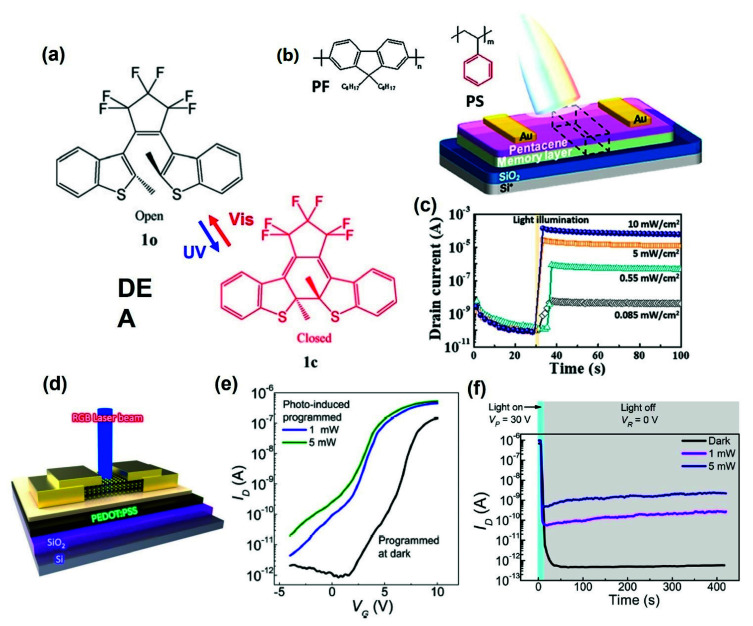
(**a**) Chemical formulae of the used DAE, in its open and closed form and structure of the devices (adapted from [104] with permission from Elsevier). (**b**,**c**) Multilevel photonic transistor memory using conjugated/insulated polymer blend electrets (adapted from [105] with permission from the American Chemical Society). (**d**–**f**) Schematic diagram of MoS_2_ flash memory with a PEDOT:PSS floating gate under light illumination and transfer curves and retention curves depending on the programming operations (adapted from [106] with permission from Nature).

Leydecker et al. reported a multistage nonvolatile optical memory thin film transistor in which the active layer is composed of an organic poly(3-hexylthiophene) (P3HT) and photochromic molecular diarylethenes (DAE) blended thin film [101]. Organic photochromic molecular DAEs have excellent thermal stability and an efficient photoisomerization process, giving them potential phototunable memory applications. The rupture and formation of the benzene ring bond of the DAE photochromic molecules cause a photochemical transformation by changing the electrical performance by irradiating the appropriate wavelength (Figure 7a) [102,103]. They reported a non-volatile device with more than 256 current levels (8-bit storage) with 3 ns laser pulses, more than 70 write/erase cycles and a data retention time of 10^7^ s. For enhanced optical operation, photochemical properties, such as absorption, refractive index, and fluorescence properties, should be considered, and physical parameters, such as highest occupied molecular orbital (HOMO), lowest occupied molecular orbital (LUMO), trap states, and charge mobility, should also be considered.

In addition, optical memory has been realized using polymer electret that responds to light. Yi et al. fabricated OFET ambipolar memory devices with a pentacene/poly(N-vinylcarbazole) (PVK)-based active layer [107]. Many excitons are generated on the pentacene layer under irradiating light and cause memory operation due to the accumulation of holes and electrons at the pentacene–PVK interface. Therefore, the device can be operated “programming/erasing” state using illumination without biasing. The OFET ambipolar memory device showed the retention times for more than 10^4^ s at holes and electron operation with on/off current ratios of 10^3^, 10^4^ respectively. Moreover, the memory window increased to 70 V, which was twice that of the OFET unipolar memory device. A conjugated polymer with high photoreactivity can be used as a photoactive component of the electret layer. On the other hand, the extended π conjugation length along the conjugated polymer backbone results in a high leakage current from the retained charges to the semiconductor. Therefore, it is also used as an electret layer in a photonic memory device through proper mixing of a conjugated polymer with an insulating polymer. Shih et al. fabricated OFET electret memory devices in which the electret layer was based on a blend of conjugated polymer poly(9,9-di-n-octylfluorenyl-2,7-diyl)(PF) and insulating polymer polystyrene (PS) (Figure 7b) [105]. The photonic memory device using the PF/PS blend electret enabled light-writing and voltage-erasing processes in a short time of one second, an on/off ratio between “Photo-On” and “Electrical-OFF” above 10^6^, and a retention time of three months. In addition, multilevel memory behavior was observed using different light sources or energy intensities of 405, 450, and 520 nm (Figure 7c).

Photo-memory devices fabricated from the synthesized electret material and perovskite were reported. Ercan et al. reported the optical and memory characteristics of a hybrid perovskite-based optical memory device synthesized with CH_3_NH_3_PbBr_3_ perovskite nanoparticles and poly(methacrylic acid) (PMAA) polymer electret materials [108]. The photoresponse behavior and the on/off current ratio increased with decreasing perovskite NPs size. The memory device without perovskite did not exhibit optical memory operations or electrical write operation, but the device with perovskite NP showed optical memory characteristics and excellent photoresponse of the perovskite. Among several polymers, a device using PMAA exhibited a high on/off switching current ratio of 10^5^.

Hong et al. presented a multifunctional optoelectronic non-volatile memory using a molybdenum disulfide (MoS_2_) semiconductor and a poly(3,4-ethylenedioxythinophene):poly(styrene sulfonate) (PEDOT:PSS) floating gate (Figure 7d) [106]. At the “program” state, it has better performance more under light than the dark. This provides 1000 times read current, a switching ratio of 2.3 × 10^7^, a memory window of 62 V, and durability for more than 1000 cycles under light (Figure 7e). Depending on whether light is illuminated, the photo-memory provided a different level of programming (Figure 7f). Therefore, a photo-activated memory that combines photodiode and memory functions could be operated.

**Table 5:** Optoelectrical Memory characteristics comparison.

Photoreaction Polymers	Wavelength (nm)	Thickness (nm)	On/Off Switching Ratio	Memory Window (V)	Retention Time (s)	Mobility (cm^2^ V^−1^ s^−1^)	[Ref]
P3HT-DAE-Me	313, 546	N/A	10^5^	N/A	10^7^	0.01	[101]
PVK	400~700	20	10^4^	70	10^4^	0.072	[107]
PF/PS blend	405, 450, 520	50–60	10^6^	18	10^5^	0.87	[105]
CH_3_NH_3_PbBr_3_	450, 530	50	10^5^	N/A	N/A	N/A	[108]
PEDOT:PSS	405, 532, 638	N/A	2.3 × 10^7^	62	2 × 10^3^	N/A	[106]

### 3.2. Neuromorphic Device

**Figure 8:**
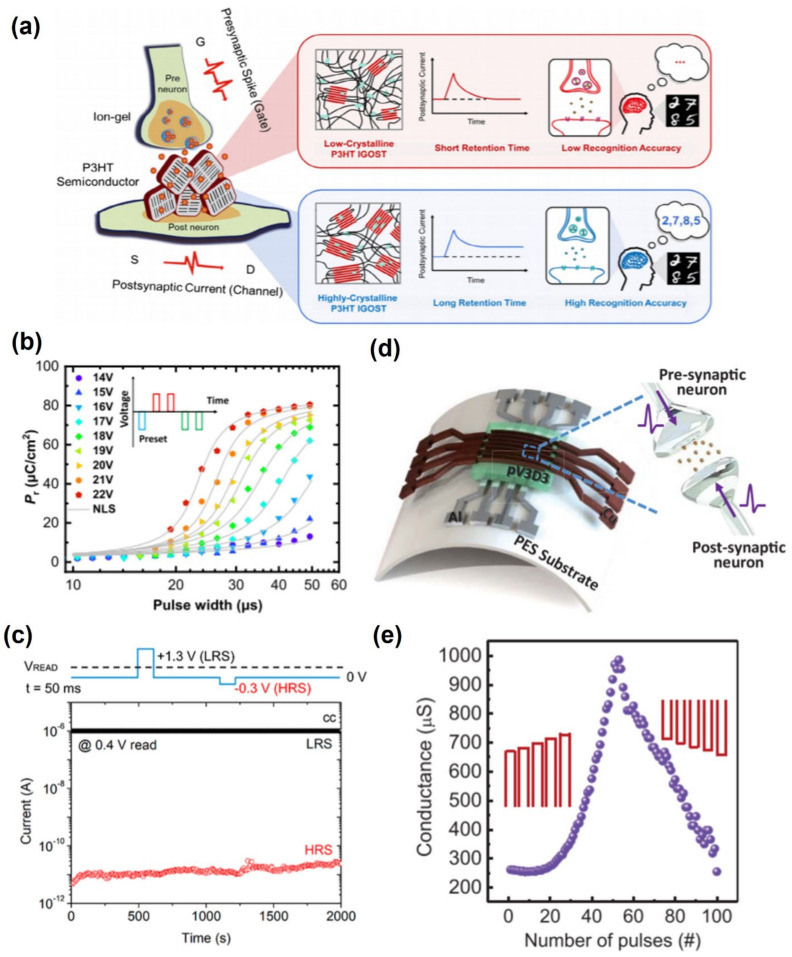
(**a**) Schematic diagram of the principle of the neuromorphic behavior using ions (adapted from [113] with permission from John Wiley and Son), (**b**) using ferroelectric polymer-based materials and its behavior according to the voltage (adapted from [114] with permission from the American Chemical Society), (**c**) ambipolar neuromorphic device and its operation mechanism (adapted from [115] with permission from the American Chemical Society), (**d**) flexible neuromorphic device using PV3d3 (adapted from [99] with permission from the American Chemical Society), and (**e**) conductance according to pulses using PV3d3 (adapted from [99] with permission from the American Chemical Society).

Neuromorphic devices are electronic devices that mimic the functions of neurons and synapses to operate electronic devices with less energy, such as human neural networks [109]. In the era of big data, neuromorphic devices will develop as the next-generation electronic devices that need to process complex data (Figure 8).

Recently, a memory device using ion gels as neurotransmitters were reported. Melianas et al. reported organic electrochemical random-access memories (ECRAMs) that were programmable under vacuum conditions at a low voltage of ±1 V using an electrically insulating polymer with common ionic liquids, 1-ethylimidazolium bis(trifluoromethylsulfonyl)imide (EIM:TFSI) [110]. Using ion gel electrolytes EIM:TFSI, the device showed linear resistive switching and faster switching down to 20 ns. The fast switching in electrochemical devices was attributed to rapidly diffusing ions, such as protons. Protons diffuse rapidly and with low activation barriers, particularly in a hydrogen-bonded network by the Grotthuss mechanism. This enables a higher dynamic range (~4x), even with fairly short ±1V 300ns write pulses, than without EIM:TFSI and superior temperature stability compared to PEDOT:PSS with a 9% increase in dynamic range. Moreover, size scaling enables faster switching down to 20 ns. Kong et al. also fabricated artificial synapses FET with amorphous indium-zinc-oxide (In-Zn-O) thin films using a P(VDF-HPF) ion-gel dielectric layer [111]. Using the same principle, Park et al. mimicked basic synaptic functions, such as EPSC, spike time-dependent EPSC, PPF, and dynamic synaptic behaviors, due to the large capacitance of the ion-gel dielectric [112].

Wang et al. reported neuromorphic devices using P(VDF-TrFE) and poly((1-vinylpyrrolidone)-co-(2-ethyldimethylammonioethyl methacrylate ethyl sulfate)) (P(VP-EDMAEMAES)) as synaptic gate dielectrics [116]. By varying the gate voltage amplitude, the synapse switches between the electrochemical and ferroelectric modes to produce short-term potentiation (STP), electrochemical long-term potentiation (LTP), and ferroelectric LTP, respectively. Furthermore, organic neuromorphic devices can be triggered in response to light and be altered into volatile and nonvolatile synaptic signals depending on the frequency, intensity, and wavelength of the light. Kim et al. reported that contact metals affect the performance of a FeFET synapse using the ferroelectric material P(VDF-TrFE) [117]. The contact barrier heights with the electrode could be modulated because of the polarization effect of the P(VDF-TrFE) film. Therefore, using the optimized device for simulating MNIST, the recognition rates of the ML-NNs had a recognition rate of 74.7%.

In addition, research on fabricating synaptic devices using a polymer as a semiconductor layer was also reported. Yu et al. used a p-type polymer, poly-(thienothiophene-co-1,4-diketopyrrolo[3,4-c]pyrrole) (PDBT-co-TT), to mimic the biological synapse behavior with data processing and nonvolatile memory capability [118]. In this device, the anion with polymers enhanced the charge retention capability of the polymer and facilitated the interchain interactions, resulting in improved memory characteristics. The suggested device can promote the development of artificial neuromorphic systems by a synaptic transistor with doped conjugated polymers.

The characteristic of pV3D3, both flexible nonvolatile memory and flexible logic-in-memory circuits have been developed for electronics applications. Jang et al. reported the transition of the operation mode in poly(1,3,5-trivinyl-1,3,5-trimethyl cyclotrisiloxane) (pV3D3)-based flexible memristor using Cu electrode working synaptic switching [119]. The set process promotes the formation of Cu filaments into the pV3D3 film without a compliance current. The reset process results in the rupture of Cu filaments due to the high currents flowing through the Cu filaments. Using a pV3D3-memristor array built on a flexible substrate, NOR gates were implemented during multiple cycles and even under bent conditions at a bending radius of 3.8 mm. This artificial neural network will encourage the development of soft neuromorphic devices. Choi et al. reported the curved neuromorphic image sensor to recognize and process massive image information [120]. This image sensor array is based on a heterostructure of MoS_2_ and poly(1,3,5-trimethyl-1,3,5-trivinyl cyclotrisiloxane) (pV3D3). The curved neuromorphic image sensor array integrated with a plano-convex lens adopting a human vision and recognition system derived a preprocessed image from a set of noisy optical inputs without redundant data storage, processing, and communications, as well as without complex optics. The photocurrent on/off switching current ratio was 11.03. Hence, it can improve efficient image acquisition and be the future way of an integrated neuromorphic sensor.

### 3.3. Flexible Devices

Flexible memory is attracting attention because of the recent advent of flexible displays and wearable devices (Table 6). In addition, it is being studied and proposed with a variety of polymer and organic materials and new structures [121,122]. This chapter discusses flexible memory: rollable [123], foldable [124], stretchable [125], fabric type, and wearable device [126,127].

**Figure 9:**
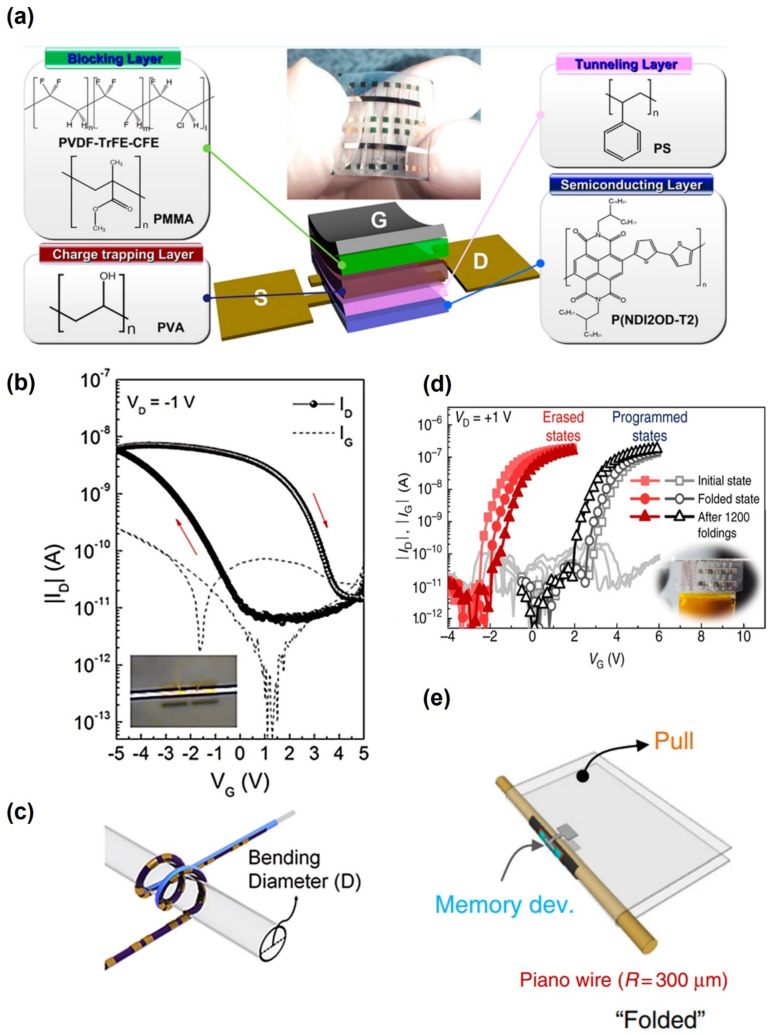
(**a**) Schematic diagram of the top-gate organic FET non-volatile memory with all polymers semiconducting, tunneling, charge trapping, and blocking layers. The chemical structure of each layer of polymers material (adapted from [128] with permission from the American Chemical Society). (**b**,**c**) Illustration of the fibriform memory coiled on a capillary tube with a diameter of 1.1 mm during measurement and transfer curve (adapted from [130] with permission from the American Chemical Society)**.** (**d**,**e**) Memory characteristics after 1200 folding cycles with a folding radius of 300 μm, which is comparable to the radius of the piano wire (adapted from [124] with permission from Nature).

Wang et al. fabricated a flexible non-volatile memory on a polyethersulfone (PES) substrate by spin-coating polymer-based layers (Figure 9a) [128]. This device was synthesized and fabricated (P(NDI2OD-T2)) as a semiconductor, polystyrene (PS) as a tunneling insulator, polyvinyl alcohol (PVA), which is an electret material, as the floating charge trapping layer, and PMMA and poly(vinylidene fluoride-trifluoroethylene-chlorofluoroethylene) P(VDF-TrFE-CFE) as the blocking insulator layers. They reported an on/off memory current ratio of 2 × 10^4^, memory window of 15.4 V, more than 100 read and write endurance cycles, and time-dependent data retention of 10^8^ s to achieve a stable and robust memory. Furthermore, devices using mechanically flexible PES substrates showed little change in performance compared to the Si substrates after 1000 bending tests at a bend radius of 5.8 mm. Li et al. fabricated a non-volatile OFET heterostructure memory devices using pentacene/N,N′-ditridecylperylene-3,4,9,10-tetracarboxylic diimide (P13)/pentacene trilayer on poly(ethylene terephthalate) PET substrate [129]. The flexible memory device implements a memory window of more than 30 V and an on/off performance of over 10^2^. The Vth showed good mechanical properties and memory performance even after 10,000 bending cycles in the mechanical bending state of a bending radius of 10 mm.

Lee et al. fabricated a flexible flash memory using a thin polymer insulator grown by ICVD (Initiated Chemical Vapor Deposition) [124]. Their non-volatile memory device was based on a C60-based organic TFT, and two iCVD processed polymer films of poly(1,3,5-trimethyl-1,3,5-trivinyl cyclotrisiloxane) (pV3D3) and poly(ethylene glycol dimethacrylate) (pEGDMA) were used as a tunneling insulator and blocking insulator, respectively. Owing to the flexibility of the iCVD-based polymer dielectric layer, devices were fabricated on 6 μm thick Mylar™ films (substrate) with an excessive bending radius (300 μm), and the device was almost foldable (Figure 9e). With a memory window of 5.5 V and an on/off ratio of 10^6^, programming and erasing functions were possible even in 1200-time folding cycles (Figure 9d).

A completely new type of flexible device that can be used in fabrics was also reported. Kang et al. reported a fibriform organic transistor memory with a nanograined organic ferroelectric film P(VDF-TrFE) solution-coated on thin, flexible Ag wires with a radius of 0.1 mm [130]. As shown in Figure 9b, a memory window of 5.6 V and an on/off ratio of 10^3^ were realized with a gate voltage of 5~−5 V. The device showed excellent switching stability of 100 cycles and stable characteristics, even when the bending tests were performed on a 1.1 mm tube (Figure 9c). The fibriform memory was stitched into a stretchable polypropylene (PP) fabric with needles, and the memory achieved stable device performance within a 5 V operating voltage in uniaxial (diagonal) deformation of 0 to 73.3% (100%) and random wrinkling harsh environment.

Stretchable devices have also been reported. It is very difficult for a stretchable device to maintain its characteristics without affecting the device under intense stress. In particular, it is difficult to use an organic material with unstable performance compared to inorganic material. On the other hand, Jung et al. fabricated stretchable organic FeFET by patterning rigid polyimide (PI) onto a polydimethylsiloxane (PDMS) elastomer substrate [125]. The PI dies were patterned using conventional photolithography and wet etching processes of elastomeric substrates, and electronic devices were fabricated on the patterned rigid PI dies. Therefore, when the substrate was stretched, the strain was mainly subjected to the stretching of the PDMS substrate, and the rigid PI die and the electronic device were subjected to a relatively small strain. Their devices obtained a memory window of 11 V, carrier mobility of 4 × 10^−2^ cm^2^ V^−1^s^−1^, and a current on/off ratio of 10^5^. These properties were reported to provide good mechanical stability and not deteriorate even at strains up to 50%.

### 3.4. Biodegradable Memory Devices

As environmental issues are emerging these days, there is an aspect that future memory semiconductor devices must also consider environmental factors. As a solution to this, efforts to develop a biodegradable polymer-based memory device are conducted. In particular, copolymerization, blending, and cross-linking methods are used for biodegradable polymer memory. Additionally, biodegradable polymer nanocomposites using nano-pillars are used [131]. However, the use of organic materials increases the biodegradability of the device but reduces performance and durability. Therefore, the compatibility of metal–semiconductor materials is important for biodegradable devices.

**Figure 10:**
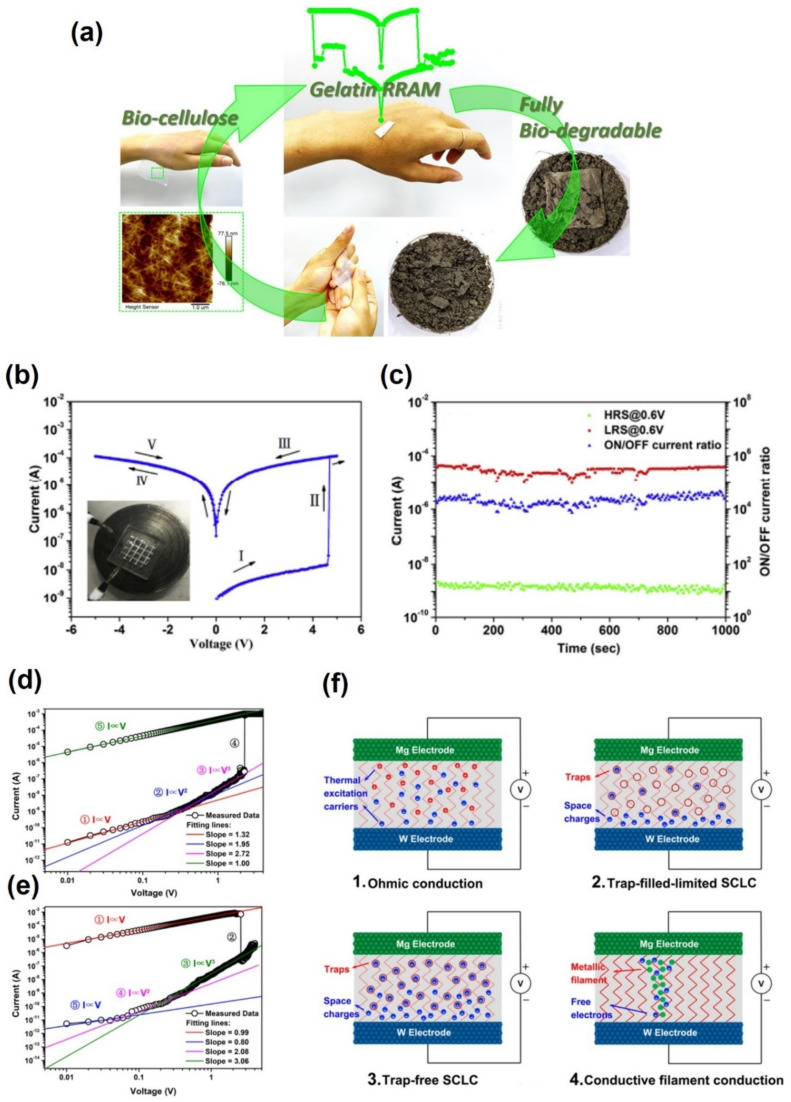
(**a**) Cellulose-based memory device that degrades in five days (adapted from [133] with permission from the American Chemical Society), (**b**) I–V curve of the resistive switching memory device and (**c**) retention and on/off ratio by time of the resistive switching memory device [132]. I–V curve of the space charge limited model according to process: (**d**) set process and (**e**) reset process [134]. Additionally, (**f**) the schematic of carrier distribution by biasing different voltage [134].

As an example of biodegradable memory development, Wu et al. suggested a memory device based on gold NP embedded alkali lignin (Au NPs: Lignin) [132]. This device has the structure polylactide substrate/bottom Al electrode/Au NPs: lignin/top Al electrode. When the carrier is trapped by the applied voltage, the electron flow path is formed so that the device has an ohmic state, and the device is switched from turn-off to -on. The electron trap between Au NP and alkali lignin is in deep-state, so detrapping is difficult even with a large reverse voltage bias. As a result, it operates as write-once-read-many times memory (WORM) memory. Furthermore, the device with a purepolylactide (PLA) substrate can be decomposited at DI water, which includes proteinase K as an enzyme that catalyzes the decomposition of L-lactyl. After soaking in enzyme solution for 5 days, surface erosion occurred, and it was decomposed into small pieces. This device shows an on/off ratio of (>10^4^), long data retention characteristics (>10^3^ s), and operates under low power (4.7 V). As an example of biodegradable memory, Huang et al. fabricated an Al/gelatin/Ag sandwich structure RRAM on a bio-cellulose (BC) film that was flexible and ductile [133]. The gelatin dielectric layer and BC film substrate were non-toxic, and environmentally friendly and fully biodegradable devices were realized. The BC film decomposed completely in the soil in just five days (Figure 10a). The device showed an on/off current ratio of over 10^4^, a low operating voltage of less than 3 V, and excellent uniformity without apparent aging at room temperature. The device was attached to pig skin and heated to approximately 37 °C to simulate human skin conditions. When measured more than 50 times, it still showed a high on/off current ratio of ~10^4^ and a long retention time of more than 7000 s. Therefore, this study highlighted applications in wearable biomedical devices, artificial electronic skin, and implantable electronic devices. Ji et al. reported an ambipolar resistive switching memory with a structure of W/silk fibroin/Mg using silk fibroin as a dielectric layer [134]. W and Mg act as inactive and active electrodes, and silk fibroin acts as a switching layer. In the set process, the abrupt binary switching behavior of Mg operates, whereas, in the reset process, the rupture of the filament is operated by an electric field. In this way, both the characteristics of the silk fibroin dielectric layer and the role of the active metal electrode are considered in the mechanism. Unlike general fibers, silk fibroin is isotropic in nature, so it is easy to expand and decomposes quickly.

Meanwhile, the electrodes can be decomposed by the reaction of Mg + 2H_2_O → M(OH)_2_ + H_2_ in DI water and 2W + 2HO + 3O_2_ → 2H_2_WO_4_ in PBS solution. The ionic of Mg^2+^ and WO_4_^2−^ can be fully metabolized in mammalian animals. In this decomposition process, all silk is dissolved within 3 h, and after 24 h of decomposition, it is completely decomposed invisibly without any residue. This device shows an on/off current ratio of 10^4^, retention of 100 cycles, memory window of 5.4 V.

## 4. Conclusions

In summary, a variety of polymer-based memories offer many advantages, as reviewed in this review. The above-revisited advances in polymer-based memory devices, including floating-gate, polymer-electret, ferroelectric, and filament-induced memory devices, have provided possibilities in the development of high-performance memory operation. Furthermore, new functionalities and their applications to optoelectrical, neuromorphic, and mechanically flexible memory integrations enabled by the polymer contributions have been considered. Different from conventional silicon or inorganic materials, polymers-based memory devices have a low process temperature (<200 °C), so the low thermal budget offers the low-cost fabrication and accordingly economical merits. Solution-processability of the polymers allows avoiding high-vacuum processes, further reducing the process’ complexity and cost.

However, more progress and efforts are needed for practical application development; polymer-based memories still have challenges, such as the following: (1) operation robustness, (2) scalability, and (3) long-term air-stability. As robust operation and scalable memory integration are the most crucial for the success of silicon-based memory devices (i.e., DRAM and Flash memory), polymer-based memory also requires research and development in the robustness and scalability aspects. To implement robust operation, attempts should be made to remove surface traps that cause operational instability. In particular, a method to overcome the low scalability of polymer-based devices is essential to make them capable of processing a lot of information in modern products. For enhanced scalability, it is essential to develop a polymer material compatible with the lithography process. In addition, the verification of air stability, which is an issue of organic materials, is also required. For practical use of polymer memory, it must be verified that it operates without degradation even after years of use and exposure to air. However, as this review introduced, the potential of polymers in next-generation memory devices is considerable enough, and we believe that in the future, polymers will be one of the keys to the development of new functional memories.

## Figures and Tables

**Table 2:** Polymer electret memory characteristics comparison.

Polymer Electret (Gate insulator)	Thickness (nm)	On/Off Switching Ratio	Memory Window (V)	Retention Time (s)	Mobility (cm^2^ V^−1^ s^−1^)	[Ref]
PS	30–35	10^6^	22.1 ± 2.8	5.2 × 10^5^	0.26 × 0.23	[55]
P*a*MS	30–35	10^6^	26.0 ± 2.7	2.2 × 10^5^	0.35 × 0.15	
P4MS	30–35	10^5^	24.1 ± 1.2	2.1 × 10^5^	0.25 × 0.07	
PVP	30–35	10^5^	17.0 ± 1.1	0.24 × 10^5^	0.21 × 0.05	
PVPyr	30–35	10^5^	21.0 ± 1.7	0.42 × 10^5^	0.12 × 0.13	
PVN	30–35	10^6^	27.8 ± 1.8	3.4 × 10^5^	0.61 × 0.05	
PVA	30–35	10^5^	Negligible	N/A	0.07 × 0.06	
P*a*MS	N/A	10^5^	24 V	10^4^	5.3 × 10^2^	[58]
PS-brush	N/A	10^4^	30 V	10^8^	0.01	[56]
TPA-PIS	~50	10^5^–10^6^	32.4 ± 1.2	10^4^	0.27 ± 0.05	[57]
TPA-PES	~50	10^5^–10^6^	43.2 ± 4.7	10^4^	0.22 ± 0.01	
TPA-PETS	~50	10^3^–10^4^	21.7 ± 1.0	10^4^	0.10 ± 0.04	
PITE(BMI-BMMD)	~50	9.0 × 10^5^	64.39	N/A	5.7 × 10^−3^	[21]
PI(APS-ODPA)	~50	9.3 × 10^4^	61.22	N/A	1.3 × 10^−3^	
PI(APS-BPA)	~50	4.6 × 10^4^	81.49	N/A	6.0 × 10^−4^	

**Table 6:** Flexible memory characteristics comparison.

Flexible Substrate	Banding Radius	On/Off Switching Ratio	Memory Window (V)	Retention Time (s)	Mobility (cm^2^ V^−1^ s^−1^)	[Ref]
PES	5.8 mm	2 × 10^4^	15.4	10^8^	3.8 × 10^−2^	[128]
PET	10 mm	10^2^	30	10^4^	0.23	[129]
Mylar™ films	300 μm	10^6^	5.5	3.2 × 10^8^	N/A	[124]
Ag wires	1.1 mm	10^3^	5.6	5 × 10^4^	9 × 10^−3^	[130]
PDMS, PI	Stretchable	10^5^	11	N/A	4 × 10^−2^	[125]

## Data Availability

Not applicable.
